# Neuroendokrine Differenzierung in Neoplasien der Prostata

**DOI:** 10.1007/s00292-025-01447-5

**Published:** 2025-07-16

**Authors:** Rainer Grobholz

**Affiliations:** 1 Medizinische Fakultät, Universität Zürich, Zürich, Schweiz; 2 Institut für Pathologie, Kantonsspital Aarau, Tellstrasse 25, 5001 Aarau, Schweiz

**Keywords:** Adenokarzinom, Androgenrezeptoren, Transdifferenzierung, Immunhistochemie, Prognose, Adenocarcinoma, Androgen receptors, Cell transdifferentiation, Immunohistochemistry, Prognosis

## Abstract

Neuroendokrine (NE) Zellen in der Prostata gehören zum diffusen neuroendokrinen System und finden sich in der normalen Prostata und in azinären Adenokarzinomen, teilweise auch mit Paneth-Zell-ähnlicher Morphologie.

NE-Zellen produzieren Peptidhormone und biogene Amine, die parakrin die Differenzierung und das Wachstum der Prostatadrüsen beeinflussen. Sie zeigen jedoch keine proliferative Aktivität und besitzen keinen Androgenrezeptor (AR).

Prostatatumoren mit NE-Differenzierung werden in 5 Gruppen eingeteilt: (1) azinäre Adenokarzinome mit partieller, nur immunhistochemisch erkennbarer NE-Differenzierung, (2) Adenokarzinome mit Paneth-Zell-ähnlicher Differenzierung, (3) NE-Tumoren/Karzinoide (NET), (4) kleinzellige NE-Karzinome (SCNEC) und (5) großzellige NE-Karzinome (LCNEC).

Die Bedeutung partieller und Paneth-Zell-ähnlicher Differenzierung in Adenokarzinomen wird diskutiert und spielt im diagnostischen Alltag eine untergeordnete Rolle. NET sind äußerst selten und scheinen sich wie NET des Gastrointestinaltraktes zu verhalten. Die SCNEC und LCNEC sind hingegen aggressive Tumoren mit wichtiger klinischer Bedeutung, da sie eine schlechte Prognose besitzen und eine aggressive Therapie nötig ist.

Das therapieassoziierte neuroendokrine Prostatakarzinom (t-NEPC) ist in der 5. Auflage der WHO-Klassifikation (2022) erstmals eine eigene Entität. Es entsteht durch Transdifferenzierung via epigenetischer Veränderungen nach Androgenentzug und weist u. a. einen AR-Verlust und eine hohe Proliferation auf. Wie bei den primären neuroendokrinen Karzinomen ist eine aggressive Therapie indiziert. Daher ist bei kastrationsresistenten progredienten Prostatakarzinomen eine Verlaufsbiopsie zum Nachweis dieses aggressiven Phänotyps zu empfehlen.

## Abstract (en)

Neuroendocrine (NE) cells in the prostate are part of the diffuse NE system and are found in both the normal prostate and acinar adenocarcinomas, occasionally exhibiting Paneth cell-like morphology.

NE cells produce peptide hormones and biogenic amines that influence the differentiation and growth of the prostate glands through paracrine signaling; however, they do not show proliferative activity and lack androgen receptors (AR).

Prostate tumors with NE differentiation are classified into five groups: (1) acinar adenocarcinomas with partial NE differentiation, detectable only by immunohistochemistry, (2) adenocarcinomas with Paneth cell-like differentiation, (3) NE tumors/carcinoids (NET), (4) small cell NE carcinomas (SCNEC), and (5) large cell NE carcinomas (LCNEC).

The significance of partial and Paneth cell-like differentiation in adenocarcinomas remains under discussion and plays a minor role in routine diagnostics. NETs are extremely rare and appear to behave similarly to NETs of the gastrointestinal tract. In contrast, SCNECs and LCNECs are aggressive tumors with important clinical relevance, as they have a poor prognosis and require aggressive treatment.

Therapy-associated neuroendocrine prostate carcinomas (t-NEPC) are recognized as a distinct entity for the first time in the WHO classification (5th edition, 2022). It arises through transdifferentiation via epigenetic changes following androgen deprivation and is characterized by AR loss and high proliferation, among other features. As with primary NE carcinomas, aggressive therapy is indicated. Therefore, a follow-up biopsy is recommended for castration-resistant progressive prostate cancer to confirm this aggressive phenotype.

## Hintergrund

Neuroendokrine (NE) Zellen in der Prostata wurden erstmals 1944 von Pretl beschrieben. Bereits damals wurde angenommen, dass diese Zellen zum diffusen neuroendokrinen System und nicht zum eigentlichen Prostatadrüsengewebe gehören [1]. Diese Annahme bestätigten Aumüller und Kollegen. Sie zeigten, dass die NE-Zellen der Prostata neurogenen Ursprungs sind und neben den Basalzellen und den sekretorischen Zellen eine eigene Zellpopulation darstellen, welche erst während der embryonalen Entwicklung in die Prostata einwandern. Dementsprechend lassen sich in den NE-Zellen auch keine spezifischen Prostatamarker finden [2]. Demgegenüber fanden andere Studien eine Koexpression von NE- und Prostatamarkern, weshalb auch ein Stammzellmodell für das Vorhandensein und die Entstehung von NE-Zellen in der Prostata diskutiert wird [3].

NE-Zellen sind mit unterschiedlicher Häufigkeit in allen Regionen der Prostata verteilt und können verschiedene Peptidhormone und biogene Amine produzieren, welche die Differenzierung und die Wachstumsregulation der Prostatadrüsen durch parakrine Mechanismen beeinflussen [4]. Sie zeigen in der Prostata keine proliferative Aktivität, besitzen keinen Androgenrezeptor (AR) und sind damit hormoninsensitiv [3].

Die Prostatatumoren mit NE-Differenzierung werden pathologisch in 5 Gruppen eingeteilt [5]. Die gemischten Tumoren (Adenokarzinome mit klein- bzw. großzelligen Karzinomen) zeigen die Veränderungen der jeweils reinen Tumoren und werden hier nicht behandelt.

1. Azinäre Adenokarzinome mit NE Differenzierung
2. Adenokarzinome mit Paneth-Zell-ähnlicher Differenzierung
3. Neuroendokrine Tumoren/Karzinoide
4. Kleinzellige NE-Karzinome (SCNEC)
5. Großzellige NE-Karzinome (LCNEC)

In der neuen 5. Auflage der WHO-Klassifikation (2022) wird das therapiebedingte neuroendokrine Prostatakarzinom (t-NEPC) erstmals als eigene Entität im Kapitel „Tumours of the prostate“ geführt, alle anderen NE-Tumoren in einem eigenen Kapitel „Neuroendocrine neoplasms“ für alle urogenitalen Organe.

## Azinäre Adenokarzinome mit partieller NE-Differenzierung

Hierbei handelt es sich um Tumoren mit der Morphologie eines azinären oder duktalen Karzinoms mit darin enthaltenen neuroendokrinen Tumorzellen (NETC), welche nur durch die immunhistochemische Darstellung von NE-Markern (Chromogranin A oder Synaptophysin) gefunden werden kann.

Das Vorkommen von NETC in azinären Adenokarzinomen der Prostata ist ein lang bekanntes Phänomen. Mehrere Studien zeigten, dass ein erhöhter intrazellulärer cAMP-Spiegel eine Transdifferenzierung von exokrinen Tumorzellen zu NETC in vitro fördert [6]. Im Rahmen dieser Transdifferenzierung verlieren die Tumorzellen die Expression des AR und zeigen keine proliferative Aktivität mehr. Dieser Effekt lässt sich u. a. durch Androgenentzug erreichen und ist nach Zugabe von Androgen-haltigem Medium reversibel [7, 8], was die Plastizität dieser Tumorzellen zeigt. Auch in vivo konnte durch Mikrodissektion und Allelsequenzierung von NETC und exokrinen Tumorzellen gezeigt werden, dass beide Zelltypen ein identisches Profil besitzen. Dies legt nahe, dass der Prozess der Transdifferenzierung auch in vivo stattfindet [8].

**Abb. 1:**
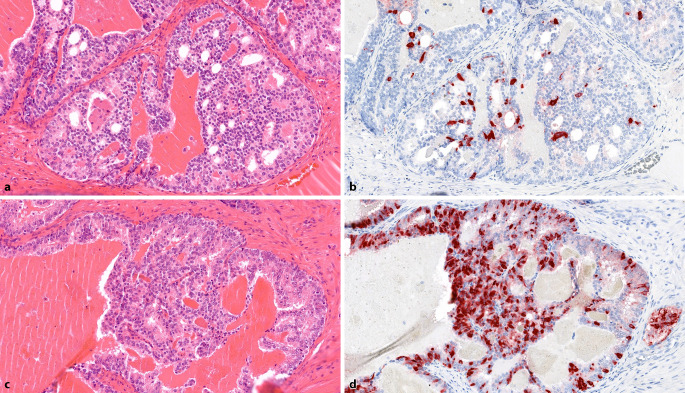
Adenokarzinom der Prostata mit partieller neuroendokriner Differenzierung. **a** Azinäres Adenokarzinom ohne evidente neuroendokrine Differenzierung. HE-Färbung, Vergr. 200:1. **b** Serienschnitt zu **a**: einzelzellig eingestreute neuroendokrine Tumorzellen. Chromogranin-A-Färbung, Vergr. 200:1. **c** Azinäres Adenokarzinom ohne evidente neuroendokrine Differenzierung. HE-Färbung, Vergr. 200:1. **d** Serienschnitt zu **c**: zahlreiche, teils in Gruppen angeordnete, neureoendokrine Tumorzellen. Chromogranin-A-Färbung, Vergr. 200:1

Das Ausmaß der NETC in unbehandelten Prostatakarzinomen (PCa) ist unterschiedlich ausgeprägt und reicht von einigen wenigen NETC bis hin zu großen Clustern (Abb. 1a–d; [9]). NETC im PCa sind spezialisierte, nichtproliferierende und AR-negative Zellen, die bioaktive Moleküle wie Serotonin und Neuropeptide produzieren und sezernieren. Diese bioaktiven Substanzen können auf die benachbarten Tumorzellen und das Mikromilieu einwirken, was deren Wachstum und die Angiogenese fördert [10, 11]. Eine einheitliche reproduzierbare Quantifizierung und damit die Bestimmung des Ausmaßes der NETC ist schwierig und wird in den Studien unterschiedlich gehandhabt. Daher ist es nicht überraschend, dass bezüglich der klinischen Bedeutung der partiellen NE-Differenzierung in PCa unterschiedliche Ergebnisse vorliegen. Die meisten Studien fanden eine Korrelation zwischen dem Ausmaß der NE-Differenzierung und dem Gleason-Score (mehr NETC in Tumoren mit höherem Gleason-Score). Hinsichtlich der Frage des biochemischen Rezidivs oder des Gesamtüberlebens war jedoch lediglich eine Signifikanz in den univariaten, nicht jedoch in den multivariaten Analysen zu finden. Aufgrund dieser fehlenden klinischen Signifikanz und der fehlenden therapeutischen Konsequenz der partiellen NE-Differenzierung in azinären PCa wird dieses Phänomen nicht mehr als eigener Abschnitt in der 5. Auflage der WHO-Klassifikation geführt. *Eine routinemäßige Bestimmung von NE-Markern (e.g. Chromogranin A oder Synaptophysin) ist in unbehandelten azinären PCa nicht empfohlen.*

## Prostatakarzinome mit Paneth-Zell-ähnlicher NE-Differenzierung

**Abb. 2:**
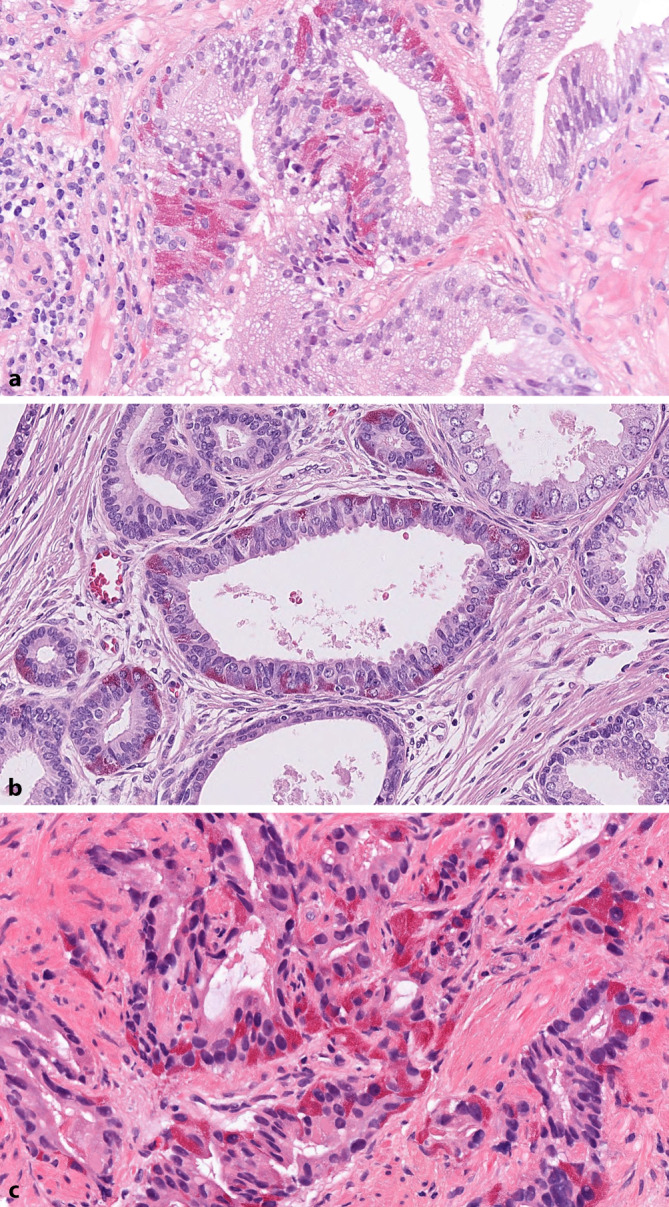
Paneth-Zell-Differenzierung mit intensivem, feingranulärem, eosinophilen Zytoplasma in tumorfreien Prostatadrüsen (**a**, HE-Färbung, Vergr. 400:1), in high-grade prostatischer intraepithelialer Neoplasie (**b**, HE-Färbung, Vergr. 400:1) und im invasiven azinären Adenokarzinom (**c**, HE-Färbung, Vergr. 400:1)

Die Paneth-Zell-ähnliche NE-Differenzierung in der Prostata findet sich gelegentlich im normalen Epithel (Abb. 2a) sowie auch in high-grade prostatischer intraepithelialer Neoplasie (Abb. 2b) und Adenokarzinomen (Abb. 2c; [12]). Diese Zellen sind gekennzeichnet durch ein intensives, feingranuläres eosinophiles Zytoplasma. Sie sind immunhistochemisch positiv für NE-Marker und negativ für Lysozym und IgA, im Gegensatz zu den Paneth-Zellen des Dünndarms [12]. Da diese Tumorzellen im Hämatoxylin/Eosin-gefärbten Schnitt leicht zu erkennen sind, ist eine immunhistochemische Darstellung nicht notwendig. Die Paneth-Zell-ähnliche NE-Differenzierung im prostatischen Adenokarzinom kann entweder in Form vereinzelter, verstreuter Zellen oder diffus in Drüsen oder Nestern vorkommen. Die Paneth-Zell-ähnlichen Zellen können in gut formierten Drüsen des Gleason-Patterns 3 vorhanden sein, aber auch ausschließlich in Zellsträngen mit unauffälliger Zytologie vorkommen. In diesen Fällen würde man ihnen bei strikter Anwendung des Gleason-Bewertungssystems ein Gleason-Pattern 5 zuweisen. In 3 Serien mit insgesamt 127 Fällen zeigte sich jedoch, dass sich das Outcome der Patienten im Wesentlichen am Gleason-Score der Tumoranteile ohne Paneth-Zell-ähnliche Differenzierung orientierte [13–15]. Die Einbeziehung Paneth-Zell-ähnlicher Areale in das Gleason-Grading führte zu einem deutlichen Upgrading, welches mit dem Outcome der Patienten nicht mehr korrelierte. Aus diesem Grund ist beim Grading dieser Fälle zu empfehlen, dass nur Areale des azinären (und/oder duktalen) Karzinoms graduiert und Areale mit Paneth-Zell-ähnlicher Differenzierung nicht berücksichtigt werden [13–15]. Dies gilt ganz besonders dann, wenn durch die Areale mit Paneth-Zell-ähnlicher Differenzierung in einem Umfeld eines ansonsten low-grade Tumors ein Upgrading zu einem high-grade Tumor erfolgen würde.

## Neuroendokrine Tumoren/Karzinoide

Echte neuroendokrine Tumoren (NET) der Prostata sind äußerst selten. Um einen NET der Prostata zu diagnostizieren und von einem Prostatakarzinom mit karzinoidähnlichen Merkmalen zu unterscheiden, sollten folgende Merkmale vorliegen:

1. nicht direkt mit einem gleichzeitigen Adenokarzinom der Prostata assoziiert sein,
2. immunhistochemisch positiv für NE-Marker und negativ für prostataspezifisches Antigen (PSA) sein,
3. den Ursprung im Prostataparenchym haben.

Wenn diese Kriterien angelegt werden, sind in der Literatur 7 Fälle dokumentiert, die diesen Kriterien entsprechen. Bei 3 Fällen handelte es sich um Patienten mit einer multiplen endokrinen Neoplasie Typ IIB im Alter von 7, 19 und 22 Jahren [16, 17], bei 3 Fällen um Patienten im Alter von 33, 34 und 37 Jahren [18, 19] und in einem kürzlich publizierten Fall um einen 67-jährigen Patienten, welcher neben einem azinären PCa einen separaten 1 mm großen NET zeigte [20]. Aufgrund der geringen Fallzahl sind Aussagen zum klinischen und biologischen Verlauf dieser Tumoren in der Prostata nur schwer vorhersagbar. In den dokumentierten Fällen lag meist ein lokal fortgeschrittenes Stadium vor, in einem Fall mit Lymphknotenmetastasen. Dennoch zeigten alle Fälle eine günstige Prognose, sodass eine Graduierung dieser Tumoren in Analogie zu neuroendokrinen Tumoren des Gastrointestinaltrakts anhand der Mitose- und Proliferationsrate (Ki67) sinnvoll erscheint. Das Grading nach Gleason kommt bei den NET nicht zur Anwendung.

Demgegenüber müssen Tumoren abgegrenzt werden, die eine karzinoid-ähnliche Morphologie aufweisen und mit einem gewöhnlichen azinären oder duktalen Adenokarzinom vergesellschaftet sind. In diesen Arealen sind die Tumorzellen in organoiden Nestern oder Inseln angeordnet, die an ein Karzinoid erinnern. Häufig haben die Tumorzellkerne jedoch noch den für PCa typischen prominenten Nukleolus und nur gelegentlich ein feingranuäres Chromatin. Zur weiteren Unterscheidung sollten daher prostataspezifische Marker (z. B. PSA) herangezogen werden, die in den echten NET negativ, in den gewöhnlichen Karzinomen mit karzinoidähnlicher Morphologie hingegen positiv ausfallen. Von der immunhistochemischen Darstellung von saurer Prostataphosphatase (PSAP) zur Abgrenzung ist abzuraten, da PSAP auch in nichtprostatischen NET exprimiert werden kann [21, 22]. Da die echten NET selten sind, gibt es bisher noch keine ausreichenden Erfahrungswerte hinsichtlich der Expression anderer prostataspezifischer Marker, wie z. B. NKX3.1, Prostein (P501S) und anderen. Tumoren mit karzinoidähnlicher Morphologie werden wie gewöhnliche PCa nach Gleason graduiert.

## Kleinzellige Karzinome (SCNEC)

**Abb. 3:**
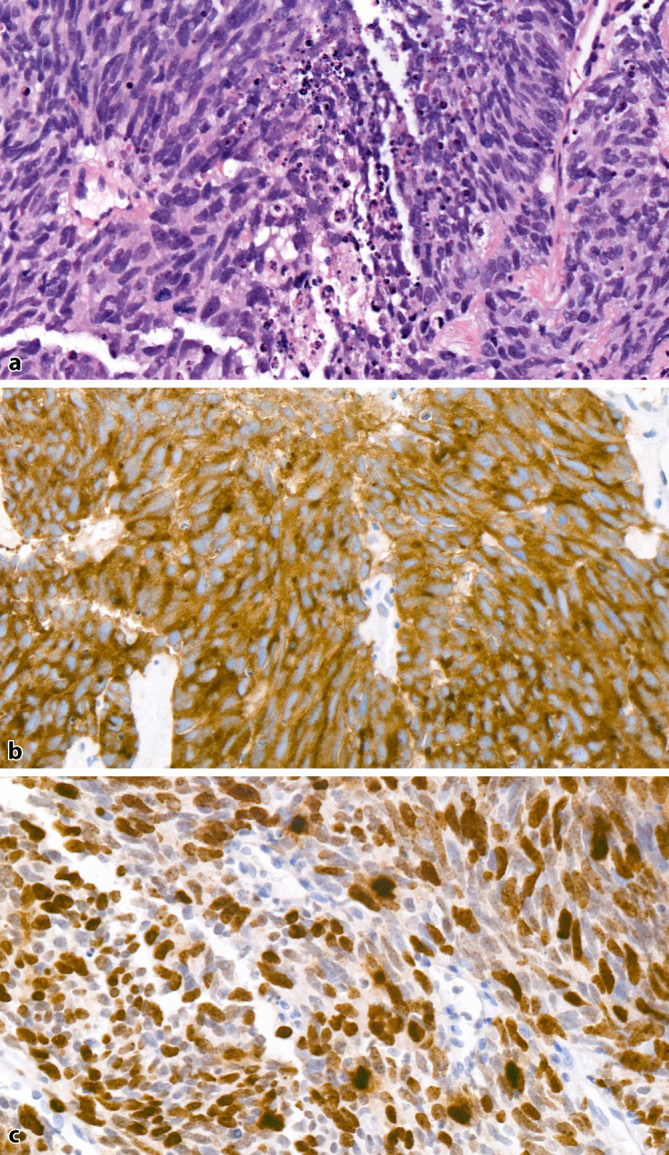
**a** Kleinzelliges Karzinom der Prostata mit wenig Zytoplasma, dunklem kondensierten Chromatin ohne prominente Nukleolen. Zahlreiche Mitosen und Apoptosefiguren, zentral nekrotischer Debris. HE-Färbung, Vergr. 400:1. **b** Kräftige durchgängige Expression von Synaptophysin in den Tumorzellen. Vergr. 400:1. **c** Hohe Proliferationsrate von ca. 70–80 %. Ki67-Färbung, Vergr. 400:1

SCNEC sind high-grade Tumoren mit charakteristischer Morphologie wie bei kleinzelligen Karzinomen der Lunge. In der Übersicht erscheint das Tumorgewebe blau und zellreich, die Tumorzellen sind klein mit wenig Zytoplasma, nukleärem Molding, häufigen Kernschmierartefakten und dunklem kondensierten Chromatin ohne prominente Nukleolen. Die Tumoren haben eine hohe Mitoserate und zahlreiche Apoptosefiguren, häufig finden sich Tumornekrosen (Abb. 3a). Wie bei den Lungentumoren kann auch hier als morphologische Variante ein intermediärer Zelltyp mit weniger dichtem Chromatin und erkennbaren kleinen Nukleolen auftreten. Immunhistochemisch sind die Tumoren in > 90 % der Fälle positiv für mindestens einen der NE-Marker Synaptophysin, Chromogranin A oder CD56 (Abb. 3b). Falls trotz typischer Morphologie die NE-Marker negativ ausfallen, kann der Nachweis einer Expression von FOXA2 oder der Verlust einer RB1-Expression hilfreich sein. Die Expression von FOXA2 kann in 75 % [23], ein Verlust der RB1-Expression in 90 % der SCNEC der Prostata gefunden werden [24]. Die Proliferationsrate (Ki67) liegt > 50 %, meist > 80 % (Abb. 3c). Ein Grading nach Gleason wird in diesen Tumoren nicht durchgeführt.

**Abb. 4:**
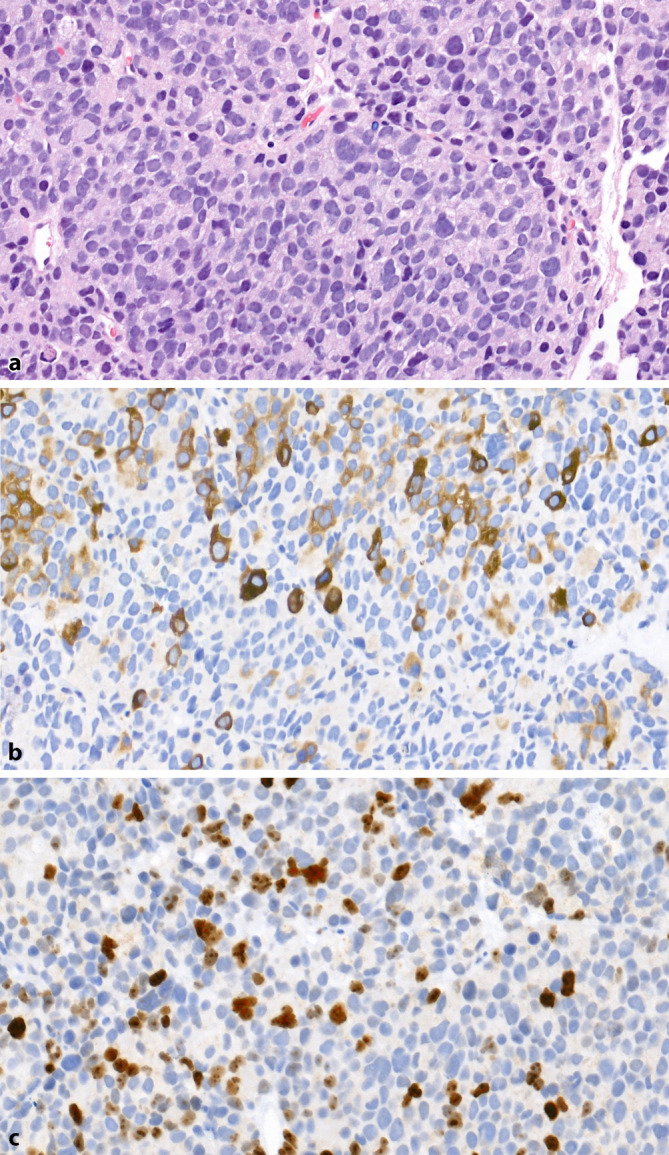
Gering differenziertes Adenokarzinom der Prostata, Gleason-Score 5 + 5. **a** Solide wachsendes Tumorgewebe, nur einzelne Tumorzellkerne sind vergrößert mit prominentem Nukleolus. **b** Nur einzelzellig findet sich eine neuroendokrine Differenzierung. Die Mehrzahl der Tumorzellen ist exokrin. Synaptophysin-Färbung, Vergr. 400:1. **c** Proliferationsrate von ca. 20 %. Ki67-Färbung, Vergr. 400:1

Primäre reine SCNEC der Prostata sind selten mit einer Häufigkeit von < 1 % und einer Inzidenz von 0,13–0,3 pro 1 Mio. Männer [25–27]. Die Ätiologie der prostatischen SCNEC ist bislang noch nicht geklärt, doch die bekannten molekularen Daten deuten darauf hin, dass diese Tumoren auch ohne Vorbehandlung aus einer Transdifferenzierung von gewöhnlichen Prostatakarzinomen entstehen und sogar eine gemeinsame Vorläuferzelle haben [28–30]. Die Abgrenzung eines SCNEC gegenüber einem gering differenzierten Adenokarzinom mit solidem Wachstum (Gleason-Score 5 + 5) kann mitunter schwierig sein, da diese Tumoren auch NE-Marker exprimieren können, jedoch keine durchgängige, sondern vielmehr eine fleckförmige Expression (Abb. 4a, b). Im Gegensatz zu den Adenokarzinomen zeigen die SCNEC immunhistochemisch eine Negativität der Prostatamarker PSA, NKX3.1 oder Prostein. Nur in etwa 17–25 % der Fälle sind in SCNEC diese Marker exprimiert und dann auch nur wenigen Zellen/Zellarealen. Eine durchgängige Expression dieser Marker in allen Tumorzellen schließt ein SCNEC weitgehend aus. Ki67 kann ebenfalls hilfreich sein, da auch gering differenzierte Adenokarzinome meist eine Proliferationsrate von < 50 % zeigen (Abb. 4c). TTF‑1 ist in ca. 50 % der SCNEC positiv, was ebenfalls hilft, ein Adenokarzinom abzugrenzen. Eine Expression von ERG als Hinweis für eine *TMPRSS2-ERG*-Fusion findet sich auch in ca. 50 % der SCNEC, welches jedoch in der Abgrenzung zum Adenokarzinom nicht hilft, da dieses eine ähnliche Positivitätsrate besitzt. Die Darstellung des AR ist ebenfalls nur begrenzt hilfreich, da dieser auch in SCNEC exprimiert werden kann [31]. Jedoch ist ein kompletter Verlust des AR aussagekräftig hinsichtlich eines möglichen fehlenden Ansprechens auf eine antiandrogene Therapie.

Klinisch werden lokalisierte SCNEC typischerweise aggressiv behandelt, oft mit multimodaler Therapie in Form von Chemotherapie und Bestrahlung, metastasierte SCNEC mit einer platinbasierten Kombinationschemotherapie ähnlich wie beim kleinzelligen Lungenkarzinom. Einige Zentren behandeln reine SCNEC nur mit Chemotherapie, während andere zusätzlich eine antiandrogene Therapie einsetzen [32]. Patienten mit dieser aggressiven Erkrankung weisen häufig viszerale Metastasen auf und weniger häufig paraneoplastische Syndrome. Die mediane Überlebenszeit liegt bei ca. 10 Monaten, ohne Metastasen bei 13 Monaten, mit Metastasen bei 8 Monaten [27].

## Therapieassoziierte neuroendokrine Karzinome (t-NEPC)

t‑NEPC sind Tumoren mit einer kompletten oder partiellen NE-Differenzierung bei zumeist kastrationsresistenten Adenokarzinomen nach Androgenentzugstherapie. Wie bereits eingangs beschrieben wurde, kann der Entzug von Androgenen eine Transdifferenzierung in einen NE-Phänotyp induzieren. Auf molekularer Ebene sind als wesentliche Faktoren für diese Transdifferenzierung die Inaktivierung (durch Mutation oder Inhibition) von *RB1* und *TP53* identifiziert. Diese doppelte Inaktivierung induziert die Umprogrammierung vom exokrinen Phänotyp zum NE-Phänotyp [33]. Die alleinige Inaktivierung eines dieser beiden Gene reicht hingegen nicht aus, um eine Transdifferenzierung herbeizuführen. Daneben finden sich auch Punktmutationen und Amplifikationen des AR, welche zusammen mit der NE-Differenzierung den AR-Verlust induzieren [30]. Darüber hinaus spielen zudem epigenetische Veränderungen eine Rolle, die die Transdifferenzierung vorantreiben: t‑NEPC zeigen eine Hypermethylierung in der Promotorregion verschiedener Gene, so etwa von „enhancer of zeste homolog 2“ (*EZH2*), welches ein Enzym codiert, das Histone modifiziert und so ganze Genregionen stilllegen kann. In t‑NEPC findet sich eine doppelt so hohe EZH2-Expression wie im noch vorhandenen Adenokarzinom [30]. Dies ist insofern interessant, da bereits erste EZH2-Inhibitoren in klinischer Erprobung stehen [34] und die Chance für eine neue zielgerichtete Therapie bieten.

In der 5. Auflage der WHO-Klassifikation wird das t‑NEPC erstmals als eigene Entität mit eigenem Kapitel aufgeführt. Mehr als die Hälfte der Tumoren entwickeln sich innerhalb der ersten 24 Monate nach Androgenentzugstherapie mit einer medianen Überlebenszeit von 7 Monaten [35]. Bei Durchführung von (palliativen) transurethralen Resektionen oder Biopsien von Metastasen fragen die Kliniker nicht selten nach einer „Anaplasie“ oder „Dedifferenzierung“. Im Klinikjargon werden diese alten Begriffe noch verwendet, gemeint ist jedoch meist die Frage nach einem t‑NEPC.

**Abb. 5:**
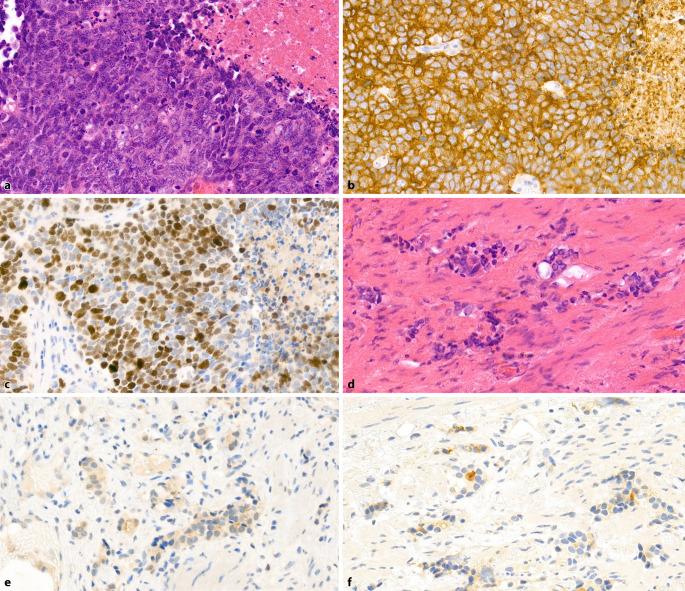
Therapiebedingtes neuroendokrines Prostatakarzinom. **a** Morphologisches Bild wie eines kleinzelligen Karzinoms mit zahlreichen Mitosefiguren und partiellen Nekrosen. HE-Färbung, Vergr. 400:1. **b** Durchgängige Expression von Synaptophysin. Vergr. 400:1. **c** Hohe Proliferationsrate von ca. 70 %. Ki67-Färbung, Vergr. 400:1. **d** Benachbart Nachweis von regressiv verändertem Adenokarzinom mit teilweise pyknotischen Zellkernen und schmalem Zytoplasma. HE-Färbung, Vergr. 400:1. **e** Fehlende Expression von Basalzellkeratinen. CK34βE12-Färbung, Vergr. 400:1. **f** Fleckförmige Expression von prostataspezifischem Antigen. PSA-Färbung, Vergr. 400:1

Morphologisch zeigen diese Tumoren ein gleichartiges histologisches Bild wie die SCNEC und sind daher praktisch nicht unterscheidbar (Abb. 5a–c). Meist finden sich noch Anteile des vortherapierten Adenokarzinoms mit entsprechenden regressiven Veränderungen, welche für die Diagnose richtungsweisend sind (Abb. 5d–f). Die Tumoren sind meist scharf voneinander abgegrenzt, nur gelegentlich finden sich auch überlappende Tumorareale. Ein Grading nach Gleason wird bei den t‑NEPC nicht durchgeführt. Auch der verbliebene Anteil des vorbestehenden Adenokarzinoms erfährt aufgrund der therapieinduzierten regressiven Veränderungen, die einen Einfluss auf die Architektur der Tumordrüsen haben, kein Grading. Die biologische und prognostische Bedeutung dieser scheinbaren Dedifferenzierung nach Androgenentzug ist unklar, weshalb ein Grading nach Gleason nicht durchgeführt wird [36].

Im klinischen Alltag ist die klinische Angabe über eine vorausgegangene Therapie wichtig und für die Diagnose entscheidend. Sollten keine klinischen Angaben vorhanden sein, kann die Suche nach regressiven Veränderungen im vorbestehenden Prostatagewebe als Indikator für eine Vorbehandlung hilfreich sein. Im Zweifelsfalle ist bei Verdacht auf eine Vorbehandlung ein Kommentar mit dem Hinweis zu empfehlen, dass mutmaßlich eine Vorbehandlung stattgefunden hat und daher kein Gleason-Grading durchgeführt wird. Sollte durch den Kliniker rückgemeldet werden, dass sicher keine Vorbehandlung stattgefunden hat, kann ein Gleason-Grading nachträglich durchgeführt werden.

## Großzelliges neuroendokrines Karzinom (LCNEC)

**Abb. 6:**
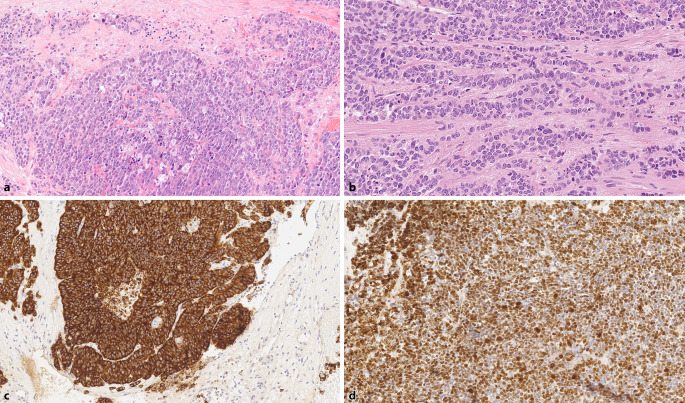
Großzellig neuroendokrines Prostatakarzinom. **a** Solide Tumorzellnester mit peripherer Palisadierung der Tumorzellen. HE-Färbung, Vergr. 200:1. **b** Strangförmiges Wachstum, Zellkerne mit feingranulärem Chromatin und kleinen Nukleolen. HE-Färbung, Vergr. 400:1. **c** Durchgängige Expression von Synaptophysin. Vergr. 200:1. **d** Hohe Proliferationsrate von > 90 %. Ki67-Färbung, Vergr. 200:1

LCNEC sind high-grade Tumoren mit einer Morphologie aus großen Tumorzellplatten mit peripherer Palisadierung, strangförmig angeordneten Tumorzellen und oft zentralen Nekrosen (Abb. 6a). Die Tumorzellen zeigen große Zellkerne mit aufgelockertem Chromatin, teilweise erkennbaren Nukleolen und abgrenzbarem Zytoplasma (Abb. 6b). Mitosefiguren sind häufig anzutreffen. Immunhistochemisch findet sich eine durchgängige Expression von mindestens einem NE-Marker (Chromomgranin A, Synaptophysin, CD56), die Proliferationsrate (Ki67) ist > 50 % (Abb. 6c, d). Die Prostatamarker PSA und saure Prostataphosphatase können exprimiert werden. In einzelnen Fällen ist auch eine Expression des Androgenrezeptors vorhanden [37]. LCNEC sind seltene Tumoren. In der Literatur sind ca. 25 Fälle beschrieben, davon 13 primäre Fälle ohne, 12 mit vorheriger antiandrogener Therapie [38]. Aufgrund der Seltenheit dieses Tumors sind umfangreiche Erfahrungen mit den Expressionsprofilen nicht vorhanden. Die Therapie ist analog der SCNEC. Die Prognose ist schlecht, in der größten Fallserie betrug die mittlere Überlebenszeit 7 Monate [39]. Wichtig ist, diesen Tumor, wie bereits beim SCNEC, von einem gering differenzierten, solide wachsenden PCa (Gleason-Score 5 + 5) abzugrenzen, da sich Prognose und Therapie dieser beiden Tumoren deutlich unterscheidet. Auch hier gelten die gleichen Unterscheidungsmerkmale wie beim SCNEC. Generell gilt, dass bei gering differenzierten PCa mit solidem Wachstum ohne eindeutig azinäre Differenzierung der prostatische Ursprung belegt bzw. ein SCNEC/LCNEC oder ein Tumor nichtprostatischen Ursprungs ausgeschlossen werden sollte.

## Fazit für die Praxis

- Die neuroendokrine (NE) Differenzierung im Prostatakarzinom (Pca) reicht von einer geringen, klinisch insignifikanten Ausprägung bis hin zu aggressiven NE-Karzinomen.
- Azinäre Adenokarzinome mit einer partiellen NE-Differenzierung werden aufgrund fehlender klinischer und biologischer Bedeutung nicht mehr geführt.
- Neuroendokrine Tumoren/Karzinoide kommen in der Prostata äußerst selten vor und unterliegen strengen diagnostischen Kriterien.
- Klein- und großzellige neuroendokrine Karzinome müssen aufgrund ihrer Aggressivität und eigenen Therapie erkannt und diagnostiziert werden.
- In der 5. Auflage der WHO-Klassifikation wird das therapiebedingte neuroendokrine PCa (t-NEPC) erstmals als eigene Entität geführt.
- Alle anderen NE-Tumoren der Prostata werden in einem eigenen Kapitel für alle urogenitalen Organe geführt.
- t‑NEPC entstehen nach Hormonentzugstherapie in kastratationsresistenten PCa und stellen hoch aggressive Tumoren dar.
- Die Abgrenzung von aggressiven neuroendokrinen Karzinomen und gering differenzierten nichtneuroendokrinen PCa gelingt durch linienspezifische immunhistochemische Marker und die Proliferationsrate.
